# A Novel Approach to Overcome Movement Artifact When Using a Laser Speckle Contrast Imaging System for Alternating Speeds of Blood Microcirculation

**DOI:** 10.3791/56415

**Published:** 2017-08-30

**Authors:** Shayan Bahadori, Tikki Immins, Thomas W. Wainwright

**Affiliations:** ^1^Orthopaedic Research Institute, Bournemouth University

**Keywords:** Medicine, Issue 126, Laser Speckle Contrast Imager, Blood flow, Microcirculation, Artifact movement, Flux, Regions of interest, Adhesive opaque patch

## Abstract

The laser speckle contrast imager (LSCI) provides a powerful yet simple technique for measuring microcirculatory blood flow. Ideal for blood dynamic responses, the LSCI is used in the same way as a conventional Laser Doppler Imager (LDI). However, with a maximum skin depth of approximately 1 mm, the LSCI is designed to focus on mainly superficial blood flow. It is used to measure skin surface areas of up to 15 cm x 20 cm. The new technique introduced in this paper accounts for alternating speeds of microcirculations; i.e. both slow and fast flow flux measurement using the LSCI. The novel technique also overcomes LSCI's biggest shortcoming, which is high sensitivity to artifact movement. An adhesive opaque patch (AOP) is introduced for satisfactory recording of microcirculatory blood flow, by subtracting the LSCI signal from the AOP from the laser speckle skin signal. The optimal setting is also defined because the LSCI is most powerful when flux changes are measured relative to a reference baseline, with blood microcirculatory flux expressed as a percentage change from the baseline. These changes may be used for analyzing the status of the blood flow system.

**Figure:**
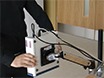


## Introduction

Laser Speckle Contrast Imaging (LSCI) is a proven non-contact, real time monitoring method to analyse blood flow microcirculation^1^,^2^,^3^,^4^,^5^,^6^,^7^. The LSCI used in this paper is moorFLPI Full Field. The measuring of blood flow perfusion in large areas at high spatial and temporal resolution using a phenomenon called "laser speckle" is one of the main advantages of this device^6^. The real-time assessment of microcirculation is done through captured patterns via a camera using scattered speckle patterns. Given that the moorFLPI LSCI is intended for clinical and physiological research, the image processing software works on the fact that high perfusion produces rapid variation in the laser speckle pattern, which is then integrated by the Charged Coupled Device (CCD) to produce an area of low contrast^8^. The contrast is quantified and the resulting flux is colour coded to produce a perfusion image^8^.

Unfortunately, the LSCI is very sensitive to environmental vibration, artifact and movement of the subject area^9^. To date this has provided challenging when alternating flow states have been studied. This paper explains details of the explicit technique outlined in a recent study^10^ where a Neuromuscular Electrical Stimulation Device was used to measure blood microcirculation when there was movement of the limb being examined.

## Protocol

The method reported was utilised in a study that received ethical approval from the Bournemouth University Research Ethics Committee on the 9th February 2016 (Reference 10571).

### 1. LSCI Set up

1. Connect the moorFLPI LSCI rear panel to its three sockets (power supply, Universal Serial Bus (USB) and IEEE1394) for the system to function.
2. Assemble the Desktop Support Arm using 4 screws with the moorFLPI LSCI turned upside down, and fixed to the support arm.
3. Rotate the mounting bracket for downward imaging when attached. NOTE: The LSCI has three controls: (1) zoom adjuster – with the imager set in a position, less magnification can be adjusted to maximum and minimum settings for small and larger fields of view respectively. To ensure repeatability, an indexing ring label is provided; (2) focus adjuster – this is dependent on the measurement distance and must be adjusted after image position has been set. To ensure repeatability, an indexing ring label is provided; and (3) a polarizer – a linear polarizing filter is available to minimize specular reflection from exposed organs – the rotating mount can be turned through 360°.
4. Install the software to control the camera. The software is divided into two modules providing a measurement and review function.

### 2. Participant Preparation

1. Ensure the assessment is performed in a temperature controlled room (22 ± 1 °C) and that the participants are seated for 10 minutes prior to testing to adapt to room temperature.
2. Avoid a strong source of artificial light and the sunlight shining on the participant or the LSCI as ambient light could affect the moorFLPI near infrared laser source operating at 785 nm. NOTE: A simple test to confirm whether ambient light levels are acceptable is by opening the imaging setup window and obstructing the laser. If the image is almost completely black then no further steps are required; if there is still too much ambient light present, further action is required.
3. Ensure the participants are relaxed throughout the assessment, with their feet flat on the ground, if seated, and avoid conversations.
4. Place 8 cm^2^ of adhesive opaque patch (AOP) (*e.g.*, Leukotape) on the skin area to mask the blood flow. This is done to account for the drawback of LSCI in terms of high sensitivity to artifact movement, and signal backscatter will be used for measurement of microcirculatory blood flow.

### 3. Microcirculation Image Measurement

1. Select 'Spatial Processing' for 25 frames per second capture at 152 x 113 pixels.
2. Choose 'Live image measurement' and adjust the position of the imager 20 cm away from the participant followed by adjusting the zoom, focus and polarizer for minimal specular reflection. The image should appear quite 'flat' and featureless.
3. Set an exposure time of 20 ms for high sensitivity to small changes and low flux.
4. Use a display rate of 25 Hz and a time constant of 0.3 s to account for rapid blood flow changes, and to achieve optimum contrast through reducing the image noise.
5. Create two equal size (2 cm^2^) regions of interest (ROI), named ROI 1 and ROI 2. Align ROI 2 so it is within the 8 cm^2^ AOP. Take care so that ROIs are not interchanging but kept close within 2-4 cm to reduce the need for re-centering if any mechanical movement results in ROI 2 no longer being in the AO area. NOTE: Blood flow measurement will be less accurate in low and high intensity areas, so it is important to have an optimal gain setting. The gain value ranges between 0 - 200. An optimal gain setting value is achieved in range of 70 - 80.
6. Perform flux measurements relative to a reference baseline. In the case of this methodology, introduce 'the rest' stage as a reference baseline. Therefore, express a 'fast' and 'slow' stage blood flow as a percentage change from a baseline, 'rest' stage.
7. Record blood flow measurement in video format and save for offline analysis using an image review module.

### 4. Offline Analysis

NOTE: The moorFLPI Image Review Software allows the opening of a video to perform analysis.

1. Calculate the mean flux within ROIs following a series of recordings of mean blood flow. ROI 1 is the real measurement of skin blood flow and ROI 2 is the backscattered laser speckle skin signal from the AOP.
2. Calculate the mean blood flow by subtracting ROI 2 from ROI 1 (skin blood flow). ROI 1 - ROI 2 = Mean Blood Flow

## Representative Results

The LSCI experimental set up is outlined in Figure 1 with functional tools identified. A typical participant preparation for a measurement of blood flow on an area of the anterior thigh is illustrated. Adjustable mounting bracket allows rotation of the LSCI for the measurement of blood flow within microvasculature of any particular skin area. Figure 2 outlines an example of a typical raw speckle image and converted speckle image with bespoke settings outlined in the protocol for measurement of blood microcirculation.

Figure 3 shows an example of skin area and placement of AOP (step 3.1), LSCI raw imaging setup (step 3.2), a live image for slow blood flow (step 3.3) and a live image for a fast blood flow (step 3.4) achieved in a continuous data recording of an alternating flux using moorFLPI LSCI. Palette color-coding setting allows differentiation between flux levels. With the standard palette of 16 colors, low flux is seen as blue, medium flux values are seen as green and high flux values are seen as orange and red. 

Skin blood flow is expressed in laser speckle perfusion units (LSPU). Figure 4 shows the graphical representation of ROI 1, ROI 2 and AOP on an area of skin. The mean blood flow is calculated in offline analysis using data from ROI 1 and ROI 2, and Equation (1).

**Figure:**
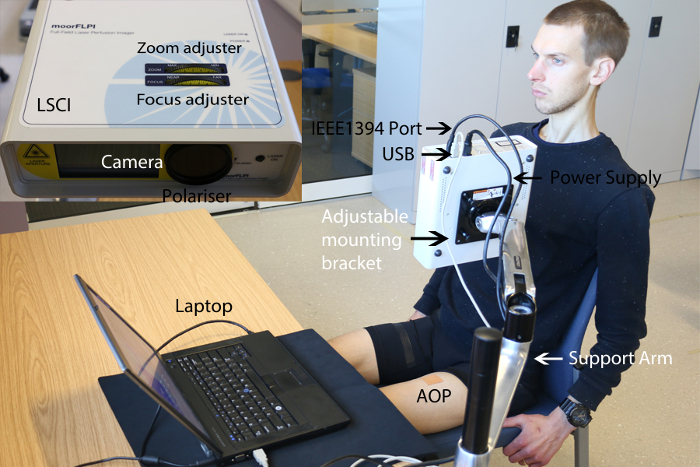

**Figure 1: moorFLPI LSCI experimental set up. **Desktop support arm, cable outputs, position controls (zoom adjuster, focus adjuster and polarizer), AOP and laptop for configuration of image setting. Please click here to view a larger version of this figure.

**Figure:**
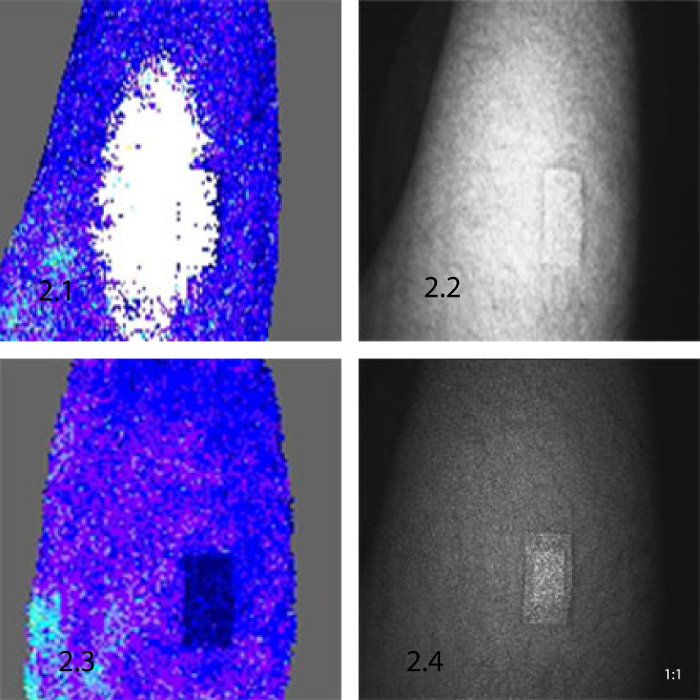

**Figure 2: Raw speckle image prior to data recording. **2.1 - 2.2) Flux and raw speckle images of a badly configured setting resulting in a high gain with poor visibility which will result in a less accurate blood flow measurement. 2.3 - 2.4) System configured as per protocol, resulting in a correct gain with maximum visibility for a reliable result. Please click here to view a larger version of this figure.

**Figure:**
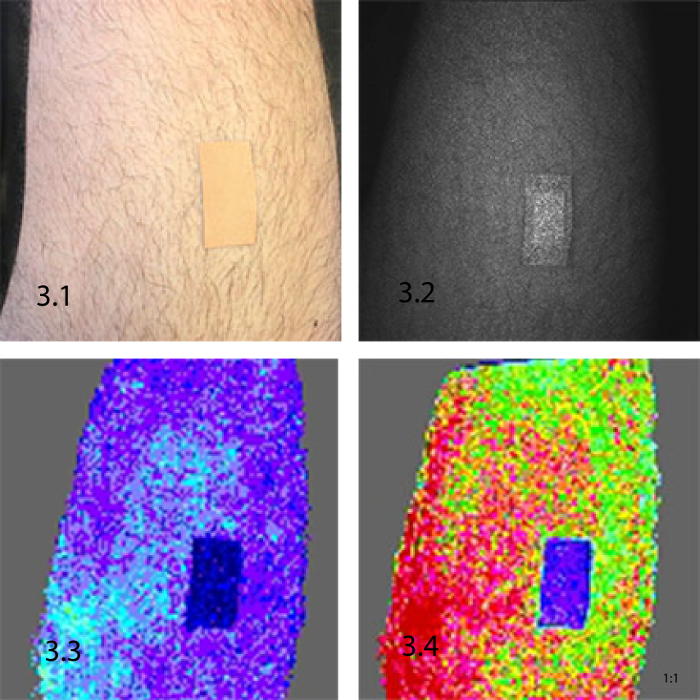

**Figure 3: An overview of setup and record measurement using moorFLPI LSCI. **3.1. An area of skin (thigh) with a 2 cm^2^ AOP to account for artifact movement. 3.2) A raw speckle 'flat and featureless' image indicating good backscatter light intensity with optimal setting. 3.3) A live image recording of a slow blood flow. 3.4) A live image recording of a fast blood flow. Please click here to view a larger version of this figure.

**Figure:**
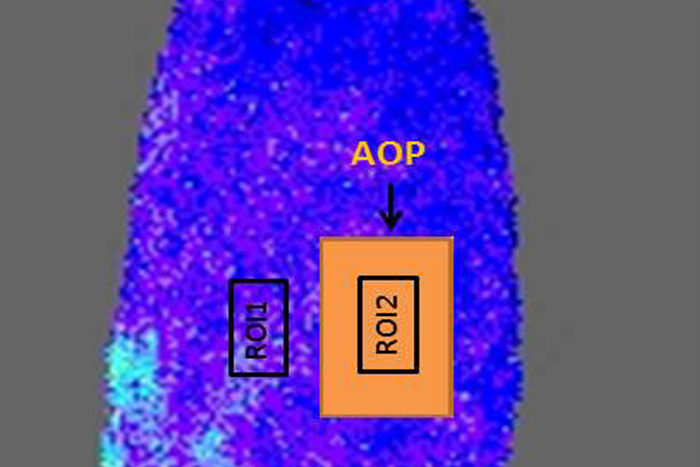

**Figure 4: Graphical representation of the ROI 1, ROI 2 and AOP layout on an area of the skin.** Please click here to view a larger version of this figure.

**Table:** 

**Rest (Baseline Reference) (LSPU)**	**Slow Blood Flow (LSPU)**	**Moderate Blood Flow (LSPU)**	**Fast Blood Flow (LSPU)**
Mean Flux - ROI 1	Mean Flux - ROI 2	Mean Blood Flow	Mean Flux - ROI 1	Mean Flux - ROI 2	Mean Blood Flow	Blood Flow Increase % from Baseline	Mean Flux - ROI 1	Mean Flux - ROI 2	Mean Blood Flow	Blood Flow Increase % from Baseline	Mean Flux - ROI 1	Mean Flux - ROI 2	Mean Blood Flow	Blood Flow Increase % from Baseline
157.9	35.1	122.8	178.5	41.6	136.9	10.9	216.9	44.6	172.3	33.5	418.9	77.5	341.4	94.2

**Table 1: Mean flux in LSPU for ROI 1 and ROI 2 at baseline, slow, moderate and fast Blood Flow. **Blood flow increase is expressed as a percentage change from a baseline stage.

**Figure:**
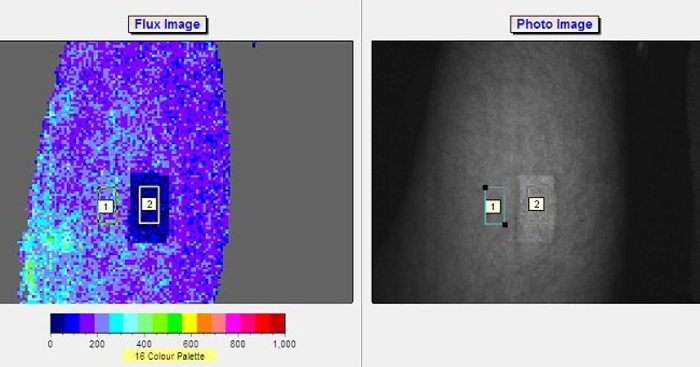

**Figure 5: Example of ROI 1 and ROI 2 positioning on a skin surface area (thigh). **A 16-color palette outlines the lavers of perfusions. Data is recorded for ROI 1 and ROI 2 in perfusion units and subtracted as explained in equation 1 for measurement of blood microcirculation. Please click here to view a larger version of this figure.

## Discussion

The aim of this study was to introduce a novel technique for the measurement and analysis of alternating speeds of blood microcirculation in a single experiment using the LSCI. The measurements can be affected by ambient light, vibration and participant movement including breathing and twitching. The steps outlined in the protocol have all been designed to minimize those effects and obtain reliable and repeatable measurements of blood microcirculation.

It is emphasized that every step within the protocol is critical for an accurate measurement of blood microcirculation, the technique introduced was discovered following sequential testing of all possible setting options and combinations available including: time constant, zoom setting, exposure time, display rate, gain and flux image palette. Results were analyzed and repeated using live video display and offline analysis to find the optimum imaging setup. This was essential as the image processing software uses the fact that high perfusion produces rapid variation in laser speckle pattern, and as a result a low contrast area of well-defined speckle is produced in the video image. Perfusion image is then created in a color-coded map of microcirculatory perfusion.

Experimental area and participation preparation is found to be essential, and this can be controlled by avoiding work near sources of daylight (window) or strong sources of artificial light, as these may interfere with the moorFLPI near infra-red laser source. The protocol also introduced an AOP as it was acknowledged that the environmental vibration and movement of participant both generate signals that are indistinguishable from blood flow. The AOP proved to be a simple yet effective choice, offering a thin but opaque, light and accessible option which had a microscopically rough surface area to avoid significant specular reflection. Preliminary research by Omarjee *et al.*^11^ highlights a potential limitation by which Leukotape creates a reflection signal amplitude different to that of the skin and varies significantly between subjects; however Mahe* et al.*^1^ found no drastic difference between participants. Although Leukotape is more accessible than other bespoke, bilayer adhesive patches, the accuracy of Equation (1) measurement could be enhanced by using an alternative AOP.

The offline analysis section highlighted the importance of sizes of ROIs, and their location within the area of interest. Initially, a bigger ROI 1, approximately 8 cm^2^, was tried which overlaid the ROI 2. This methodology soon became un-reliable due to artifact movement resulting in the ROI 2 moving and the live experiment had to be stopped in order to re-center the ROI 2. Another short coming was that due to ROI 1 overlaying the AOP, the mean flux no longer took the area under AOP into an account, as there was no longer a backscattered signal. This meant a large section of blood microcirculation was being overlooked and therefore resulting flux data were not correct. Therefore, a methodology in which two ROIs of 2 cm^2^, with an AOP of 8 cm^2^ and no interaction between ROI 1 and ROI 2 (but kept within 2-4 cm of each other), provides a reliable and repeatable analysis technique for measurement of blood microcirculation.

## Disclosures

The authors have nothing to disclose.
